# Clinical Lectures on Diseases of the Eye

**Published:** 1865-05

**Authors:** E. L. Holmes

**Affiliations:** Chicago, Lecturer on Diseases of the Eye and Ear in Rush Medical College, and Surgeon of the Chicago Charitable Eye and Ear Infirmary


### CLINICAL LECTURES ON DISEASES OF THE EYE.

By E. L. HOLMES, M. D., of Chicago,

*Lecturer on Diseases of the Eye and Ear in Rush Medical College, and Surgeon of the Chicago Charitable Eye and Ear Infirmary.*

### GLAUCOMA.

*Gentlemen:*—Glaucoma in its strict sense, as now defined by the results of comparatively recent investigations, is an inflammation of all the internal tissues of the globe, influenced if not caused by a congestion of these tissues, which is always accompanied by an abnormal hardness of the globe and a depression in the papilla of the optic nerve.

The subjective symptoms are usually pain, indistinctness of vision, and the appearance of colored rings around luminous objects. The amaurotic symptoms often commence with a diminution in the extent of the field of vision, and with a marked degree of far-sightedness. These subjective symptoms vary much in severity in different individuals. The pain for instance, may be slight, or it may be agonizing beyond description, especially in acute cases.

By simple inspection the sub-conjunctival vessels are usually found congested, the pupil dilated, although if iritis be present, it may be contracted; the iris discolored and lying nearly if not quite in contact with the cornea; later the aqueous and vitreous humors and lens become cloudy, often giving a peculiar green color to the pupil.

One of the most important objective symptoms, is the abnormal hardness of the globe, best observed by gently pressing the ends of the two fore-fingers alternately upon the closed upper lid. It is difficult to detect this hardness when there is much tenderness or swelling of the lids or conjunctiva. It will be advantageous for you to form the habit of examining normal eyes, to enable you better to detect different degrees of unnatural hardness or softness of the globe. The cornea becomes less sensitive than normal, as shown by rubbing foreign substances over its surface.

By means of the ophthalmoscope, two very important changes can be observed, before the humors of the eye have become opaque. The papilla of the optic nerve instead of being a plain disk is found to be cupped. The central vessels of the retina, especially the artery, present a peculiar pulsating motion, synchronous with the action of the heart.

Other changes which may be observed in the posterior portion of the eye are not peculiarly characteristic of glaucomatous disease. Before all these symptoms have become marked there are often certain premonitory indications of the approaching disease, such as severe headache, fugitive pains in and about the eyes, temporary congestion of the deeper conjunctival vessels, periodic attacks of dimness of vision, with the appearance of faint-colored rings around a lighted candle. This last symptom, when not dependent upon the presence of mucous on the cornea, should always place the practitioner on his guard in reference to a prognosis.

By recalling to mind a few points in the anatomy and physiology of the choroid and iris, you will readily understand the causes of these symptoms. The inflammation of the choroid, which either covers or encloses the nerves and vessels, supplying the iris and ciliary processes, interferes with the normal action of these vessels and nerves not only directly by the influence of the inflammation in the choroid itself, but also secondarily by the pressure upon them, produced by the increased contents of the globe, from serous exudation. Hence the ciliary muscle is paralyzed, and the patient cannot accommodate the eye for near objects. The ciliary branches of the fifth pair of nerves supplying the cornea, become paralyzed, and the cornea loses its sensibility. The pupil is dilated, since the circular muscular fibers of the iris become paralyzed before the radiating fibres. If the pupil is oval or irregular, it will be found drawn towards the portion of the choroid, most seriously affected. The serous exudation just mentioned forces the lens and iris forward, sometimes obliterating the anterior chamber. For the same reason the optic nerve is forced more or less through the opening in the sclerotic, giving it the peculiar cupped appearance. The ordinary forces which cause the blood to flow in a continuous stream through the central vessels of the retina, are insufficient to carry the blood through them when pressed upon by the increased quantity of fluids in the eye. The contraction of the heart alone is able to fill the artery, hence the blood passes into the globe with a sudden pulsation.

There is a form of choroidal inflammation which merits your attention, as often causing increase of the intraocular fluids and consequent tenseness of the globe with concavity of the papilla of the optic nerve. This disease also is sometimes termed glaucomatous. It should be remembered, however, that it is an inflammation of the *choroid*, which is liable to extend to the retina and other tissues, while glaucoma is from the very beginning a congestion or inflammation of the choroid, iris and retina together.

This form of simple choroiditis is often caused by injuries, or by a syphilitic or rheumatic diathesis. There seems to be a tendency to the disease in some patients suffering from serious diseases of the heart, lungs, or liver, and in females at the period of final cessation of the menses. True glaucoma may depend in a measure upon these same causes, but there seems to be two other conditions of the eye necessary—a calcarious or atheromatous condition of the vessels of the orbit, of the base of the brain, and even of the globe itself—and a hardened, unyielding condition of the sclerotic. Comparatively recent investigations would lead us to believe that true glaucoma never occurs without a previous change in the vessels as just mentioned. Hence the disease is, like the affection of the vessels, peculiar to patients past middle life; and as the vessels of both orbits are almost invariably affected, so glaucoma, if it attacks one eye, is almost inevitably to appear sooner or later in the other eye. The condition of the orbital vessels explains why the congestion in the globe should not be confined to the choroid, as in choroiditis.

It is worthy of notice that choroiditis with serous exudation, although it may occur in the aged, is quite common in patients below middle age. It often causes in such patients a dilatation of a portion of the sclerotic (staphyloma scleroticæ) or a general dilatation as in Hydrophthalmia, and yet the papilla may remain almost normal as regards any appearance of depression, as if this last symptom depended in a measure upon an abnormal induration of the tissue of the sclerotic. The disease may be very sudden and acute, with most violent symptoms, or it may be chronic, with symptoms so mild as scarcely to attract the patient's notice for some time. Although the symptoms may occasionally subside without treatment, they are sure to reappear. Loss of vision and atrophy of the globe are the inevitable results of the disease. Medical treatment is absolutely without permanent benefit.

Surgical interference, either in the operation of Iridectomy, as recommended by Græfe, or of section of the ciliary muscle, as proposed by Hancock, is the most reliable means now known for relieving the disease. The application of Iridectomy in the treatment of glaucomatous disease was one of the most brilliant triumphs in operative surgery. Patients suffering the most agonizing pains and totally blind, have been relieved of their pain and restored to sight. If, however, we take the aggregate experience of oculists, throughout the world, we must admit that the operation has not proved of so much benefit in so large a proportion of cases as its friends seemed at first to anticipate. The operation appears to relieve pain in a greater number of cases than it restores vision. The full benefits of the operation are mostly obtained in the earlier periods of the disease, and in acute rather than chronic cases. The operation of Iridectomy in glaucoma is similar in many respects to that for artificial pupil. It is important that at least one-sixth of the iris be fully separated from its ciliary attachments. To accomplish this is sometimes a matter of difficulty. Two punctures through the cornea near together have been recommended as facilitating this part of the operation. When it is remembered that the iris and lens are often in direct contact with the cornea, the difficulty of performing the operation without injury to the lens will be readily appreciated.

The division of the ciliary muscle is accomplished by passing the point of a cataract knife, the back of the instrument being turned to the centre of the pupil, through the sclerotic about a line from the periphery of the cornea, till an incision has been made two or three lines in length. The edge of the lens should be avoided.

The precise manner in which these operations prove beneficial are not well understood. For different theories and for the discussion relative to the comparative utility of these two operations, I must refer you to the original works of Græfe or their translations, the later foreign journals and hand-books on Diseases of the Eye. Possibly the articles in Braithwaite of the past few years and the monograph of Dr. Keyser, of Philadelphia, would be as useful works for you to read as any you can readily obtain.

